# Prognosis of LSPD versus TIPS for the treatment of esophagogastric variceal bleeding in cirrhosis

**DOI:** 10.1007/s00464-024-10729-7

**Published:** 2024-03-04

**Authors:** Biao Chen, Jingxuan Wang, Weiyong Sheng, Bingqing Ma, Peng Xu, Xing Cheng, Weiyi Cheng, Chengjun Cai, Guoliang Wang, Wenming Pan, Chidan Wan, Chuansheng Zheng, Ping Cheng, Jinxiang Zhang

**Affiliations:** 1 Department of Emergency Surgery, Tongji Medical College, Union Hospital, Huazhong University of Science & Technology, Wuhan, 430022 Hubei China; 2 Wisdom Lake Academy of Pharmacy, Xi’an Jiaotong-Liverpool University, Suzhou, 215123 Jiangsu China; 3 Department of Cardiac Surgery, Wannan Medical College, Yijishan Hospital, Wuhu, 241000 Anhui China; 4 Health Management Center, Tongji Medical College, Union Hospital, Huazhong University of Science & Technology, Wuhan, 430022 Hubei China; 5 Department of Hepatobiliary Surgery, Tongji Medical College, Union Hospital, Huazhong University of Science & Technology, Wuhan, 430022 Hubei China; 6 Department of Interventional Radiology, Tongji Medical College, Union Hospital, Huazhong University of Science & Technology, Wuhan, 430022 Hubei China

**Keywords:** Portal hypertension, Esophagogastric variceal bleeding, Laparoscopic splenectomy combined with pericardial devascularization, Transjugular intrahepatic portosystemic shunt

## Abstract

**Background:**

This study aimed to compare postoperative complications in patients with esophagogastric variceal bleeding (EVB) who underwent laparoscopic splenectomy combined with pericardial devascularization (LSPD) versus transjugular intrahepatic portosystemic shunt (TIPS) procedures.

**Methods:**

A retrospective collection of medical records was conducted from January 2014 to May 2020 at Union Hospital, Tongji Medical College, Huazhong University of Science and Technology. The study included patients from the departments of trauma surgery, interventional radiology, and general surgery who were diagnosed with EVB caused by portal hypertension and treated with LSPD or TIPS. Follow-up data were obtained to assess the occurrence of postoperative complications in both groups.

**Results:**

A total of 201 patients were included in the study, with 104 cases in the LSPD group and 97 cases in the TIPS group. There was no significant difference in the 1-year and 3-year post-surgery survival rates between the TIPS and LSPD groups (*P* = 0.669, 0.066). The 3-year survival rate of Child–Pugh B patients in the LSPD group was higher than TIPS group (*P* = 0.041). The LSPD group also had a significantly higher rate of freedom from rebleeding at 3-year post-surgery compared to the TIPS group (*P* = 0.038). Stratified analysis showed no statistically significant difference in the rebleeding rate between the two groups. Furthermore, the LSPD group had a higher rate of freedom from overt hepatic encephalopathy at 1-year and 3-year post-surgery compared to the TIPS group (*P* = 0.007, < 0.001). The LSPD group also had a lower rate of severe complications at 3-year post-surgery compared to the TIPS group (*P* = 0.020).

**Conclusion:**

Compared to TIPS, LSPD does not increase the risk of mortality and rebleeding, while demonstrating fewer complications. In patients classified as Child–Pugh A and B, the use of LSPD for treating EVB is both safe and effective.

**Supplementary Information:**

The online version contains supplementary material available at 10.1007/s00464-024-10729-7.

Currently, liver disease is one of the leading causes of death worldwide. The emergence of complications from portal hypertension indicates a transition from the compensated stage to the decompensated stage of liver cirrhosis, which leads to an accelerated disease progression and increased hospitalization rates, thereby increasing the burden on healthcare systems. Statistics show that approximately 50% of liver cirrhosis patients develop gastroesophageal varices (GOV) due to portal hypertension [1]. Digestive tract bleeding caused by GOV is a significant cause of mortality in patients with decompensated liver cirrhosis. Despite decades of efforts, the mortality rate of acute esophageal gastric variceal bleeding (AEVB) remains high, ranging from 10 to 20% [2, 3]. Endoscopy, transjugular intrahepatic portosystemic shunt (TIPS), and devascularization procedures are the main treatment options for AEVB and the primary preventive measures for recurrent esophagogastric variceal bleeding (EVB). Existing studies have provided varying degrees of affirmation for these three prevention strategies, but there are certain differences in their selection [4–8]. TIPS and devascularization procedures are primarily used for bleeding treatment after the failure of endoscopy combined with medication. Both TIPS and devascularization procedures are the main treatment options for acute bleeding control and prevention of rebleeding in AEVB, particularly in China, where laparoscopic splenectomy combined with pericardial devascularization (LSPD) is widely employed in the treatment of EVB. Recent studies have shown that TIPS is superior to endoscopic variceal ligation (EVL) combined with *β*-blockers as secondary prevention for variceal rebleeding, but TIPS does not improve patient survival rates [6, 7]. In a propensity score matching study, postoperative rebleeding rates were lower in patients treated with devascularization procedures than in the endoscopy group [9]. Another retrospective study demonstrated that open splenectomy and esophagogastric devascularization (OSED) is superior to TIPS in the treatment of recurrent variceal bleeding and prevention of rebleeding in patients with Child–Pugh A and B liver function [8]. Although endoscopic treatment has advantages, such as ease of operation, minimal trauma, and high initial hemostasis rates, the probability of rebleeding after endoscopic treatment is relatively high, with a 1-year rebleeding rate of up to 45% [6]. TIPS is currently the internationally recommended treatment option for rebleeding and its therapeutic value is acknowledged, but it may result in serious complications, such as hepatic encephalopathy and stent occlusion. While the use of covered TIPS stents reduces the risk of stent thrombosis, the incidence of overt hepatic encephalopathy (OHE) can still exceed 32% [10]. Currently, surgical treatments lack comprehensive evaluation based on multicenter, randomized controlled trials, which hinders the establishment of their definite role [11].

To comprehensively understand the current status of surgical treatment for portal hypertension, the Chinese Hepatopancreatobiliary Association and the China Portal Hypertension Alliance (CHESS) have jointly initiated a study on the surgical treatment of portal hypertension in China to establish and improve the management system for surgical treatment of portal hypertension. Currently, there is a lack of research data elucidating the differences in efficacy between LSPD and TIPS. This study aims to collect case data of patients treated with LSPD and TIPS and conduct a prospective follow-up to compare the prognosis differences between the two treatment modalities.

## Materials and methods

### Patients

This retrospective single-site study was conducted at Union Hospital, Tongji Medical College, Huazhong University of Science and Technology. Clinical data were collected from May 2021 to December 2022. The analysis included 97 patients with EVB treated with TIPS and 104 patients with EVB treated with LSPD. This study was approved by the Medical Ethics Committee of Union Hospital, Tongji Medical College, Huazhong University of Science and Technology (Approval No. 2023 0234). All informed consent for this study was obtained through telephone follow-ups. The inclusion criteria were as follows: (1) Age between 22 and 75 years; (2) Diagnosis of portal hypertension due to cirrhosis with esophagogastric variceal bleeding; (3) Liver function classified as Child–Pugh class A or B. The exclusion criteria were as follows: (1) Pregnant or lactating women; (2) Presence of organ failures such as severe pulmonary disease, renal failure, cardiac dysfunction, and multi-organ failure/sepsis; (3) Patients with Child–Pugh class C; (4) Previously received two or more types of treatment or combination therapy; (5) Hypercoagulable state not related to liver disease [12]; and (6) Patients with unstable hemodynamics after EVB. All patients underwent necessary medical treatments before surgery, including endoscopic therapy for persistent acute bleeding, assessment of coagulation function, evaluation of albumin levels, correction of anemia, and improvement of liver function. These patients were given the option of either TIPS or LSPD treatment, with the specific approach suggested by the doctor and determined through consultation between the patient and their family.

### The TIPS group

TIPS can be used in EVB patients with Child–Pugh score < 14, and for patients with severe varices under contrast, simultaneous variceal embolization can be performed. In the case of concomitant splenomegaly, splenic artery embolization (SAE) can also be performed. All patients received 8 mm TIPS covered stent. Supplemental File 1 provides a detailed description of the procedure. Starting from 3 days after surgery, low-molecular weight heparin is used to prevent thrombosis. Follow-up examinations of the portal vein system using ultrasound are performed at 2 weeks, 1 month, 3 months, and 6 months after surgery to assess stent patency. Regular blood ammonia monitoring is conducted postoperatively, and medications such as lactulose are administered to promote bowel movement. Aspartate ornithine is used to facilitate ammonia metabolism and prevent hepatic encephalopathy caused by elevated blood ammonia levels.

### The LSPD group

LSPD is indicated for the following patients: (1) those with moderate to severe variceal bleeding who experience recurrent bleeding despite medical or TIPS treatment; (2) patients who have concurrent splenomegaly and hypersplenism and in whom both endoscopy and TIPS treatment cannot be simultaneously corrected. Postoperatively, prophylactic antibiotics are administered for 3–5 days to prevent infection, while low-molecular weight heparin is initiated on the third day after surgery as thromboprophylaxis. For patients with a postoperative platelet count exceeding 500 × 10 [9] /L, aspirin is used as an antiplatelet agent for a continuous duration of 3 months. During this period, regular follow-up examinations using portal vein ultrasound are conducted to assess the occurrence of thrombosis, and if necessary, the duration of aspirin use can be adjusted accordingly. Supplemental File 1 provides a detailed description of the procedure.

### Data collection and follow-up

All patients underwent laboratory, radiologic, and endoscopic examinations. Baseline data included age, gender, BMI, hypertension, diabetes, etiology of liver cirrhosis, ascites severity, location of varices, previous bleeding frequency, portal vein thrombosis, white blood cell (WBC), hemoglobin (HGB), platelet (PLT), alanine aminotransferase (ALT), aspartate aminotransferase (AST), total bilirubin (TBIL), direct bilirubin (DBIL), gamma-glutamyl transpeptidase (GGT), alkaline phosphatase (ALP), creatinine (Cr), international normalized ratio (INR), and prothrombin time (PT). The Child–Pugh grade and model for end-stage liver disease (MELD) scores were recorded. The occurrence of postoperative complications was recorded for all patients. Follow-up data on post-discharge complications were obtained. Postoperative complications were recorded in terms of onset time and treatment outcomes and follow-up was completed for all patients in April 2023. All complications were graded using the Clavien–Dindo Surgical Complication Grading System, with Clavien–Dindo grade ≥ III defined as a serious complication [13]. Supplemental File 1 provides a detailed description of the surgical procedure.

### Statistical analysis

Data analysis and graphing were performed using IBM SPSS version 29.0 and GraphPad Prism version 10.0. Continuous variables were described as mean ± standard deviation (SD) or median (interquartile ranges, IQR) and compared using Student’s t test or Mann–Whitney U test. Categorical variables were presented as percentages and compared using Pearson’s chi-square test or Fisher’s exact test. Time-to-event curves between the two groups were compared using the Log-Rank test, and Kaplan–Meier curves were plotted. Two-sided *P*-value < 0.05 were considered statistically significant.

## Results

### Baseline characteristics

**Fig. 1:**
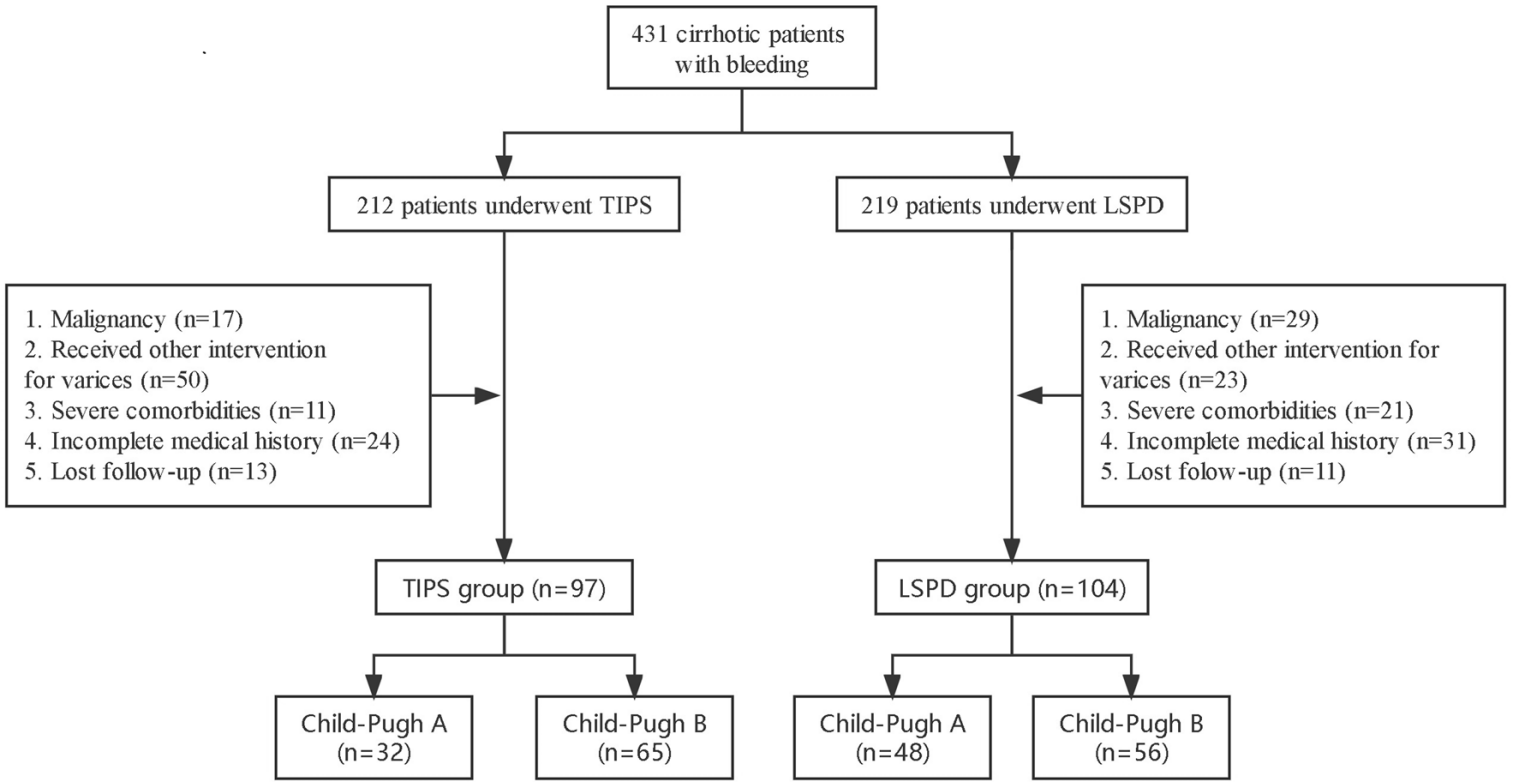
Flowchart of patient selection process

From January 2014 to May 2020, 212 patients with EVB were treated with TIPS, while 219 patients with EVB underwent LSPD. Among them, 97 patients in the TIPS group and 104 patients in the LSPD group were included in the analysis, as shown in the flowchart (Fig. 1). There were no significant differences in baseline characteristics between the two groups (Table 1). The median follow-up duration was 1138 days in the TIPS group and 1715 days in the LSPD group.

**Table 1:** Baseline characteristics of the two groups

Characteristics	TIPS (*n* = 97)	LSPD (*n* = 104)	*P*
Age (year)	54.8 ± 11.6	52.7 ± 10.3	0.168
Male sex, (%)	60 (61.9)	68 (65.4)	0.709
BMI	22.3 (19.9, 26.6)	23.4 (20.6, 26.7)	0.287
Hypertension, (%)	17 (17.5)	17 (16.3)	0.972
Diabetes, (%)	16 (16.5)	17 (16.3)	0.977
*Etiology, (%)*			0.796
Hepatitis B	67 (69.1)	73(70.2)	
Hepatitis C	10 (10.3)	14 (13.5)	
Alcoholic hepatitis	4 (4.1)	6 (5.8)	
Others	16 (16.5)	11 (10.5)	
Emergency, (%)	19 (19.6)	12 (11.5)	0.114
MELD score	9.9 (8.9, 11.2)	10.1 (9.3, 11.4)	0.135
WBC (× 10^9^/L)	3.3 (2.2, 5.1)	3.0 (2.4, 4.3)	0.631
HGB (× 10^9^/L)	77.0 (68.0, 86.0)	76.0 (68.0, 86.0)	0.775
PLT (× 10^9^/L)	66.0 (43.5, 115.0)	68.5 (51.3, 84.8)	0.664
ALT (U/L)	25.0 (16.0, 39.0)	21.0 (14.0, 34.0)	0.116
AST (U/L)	30.0 (24.0, 41.5)	28.0 (20.3, 38.3)	0.106
TBIL (umol/L)	16.4 (12.4, 23.5)	18.9 (13.2, 22.2)	0.618
DBIL (umol/L)	7.3 (5.3, 10.9)	8.2 (6.0, 10.4)	0.479
GGT (U/L)	26.0 (19.0, 46.0)	28.0 (18.0, 46.8)	0.648
ALP (U/L)	67.0 (51.5, 89.5)	69.0 (56.0, 92.0)	0.370
Cr (umol/L)	66.2 (56.5, 75.6)	66.2 (56.1, 78.1)	0.937
INR	1.3 (1.2, 1.4)	1.3 (1.2, 1.4)	0.134
PT (s)	15.8 (15.2, 17.2)	16.6 (15.3, 17.6)	0.083
*Ascites, (%)*			0.140
No ascites	33 (34.0)	23 (22.1)	
Mild ascites	37 (38.2)	51 (49.0)	
Moderate and severe ascites	27 (27.8)	30 (28.9)	
*Location of varices, (%)*			0.381
Esophageal varices only	10 (10.3)	17 (16.4)	
Gastric varices only	5 (5.2)	7 (6.7)	
Esophageal and gastric varices	82 (84.5)	80 (76.9)	
Bleeding frequency (1/2/3/4/6)	33/37/21/5/1	43/37/16/8/0	0.496
Thrombosis of portal vein system, (%)	15(15.5%)	19(18.3%)	0.596
*Child–Pugh grade, (%)*			0.078
A (5–6)	32 (33.0)	48 (46.2)	
B (7–9)	65 (67.0)	56 (53.9)	
Treatment, (%)			
TIPS only	44 (45.4)	–	
TIPS + SAE	41 (42.3)	–	
TIPS + variceal embolization	5 (5.2)	–	
TIPS + SAE + variceal embolization	7 (7.4)	–	
LSPD	–	104 (100)	

### Survival analysis

**Fig. 2:**
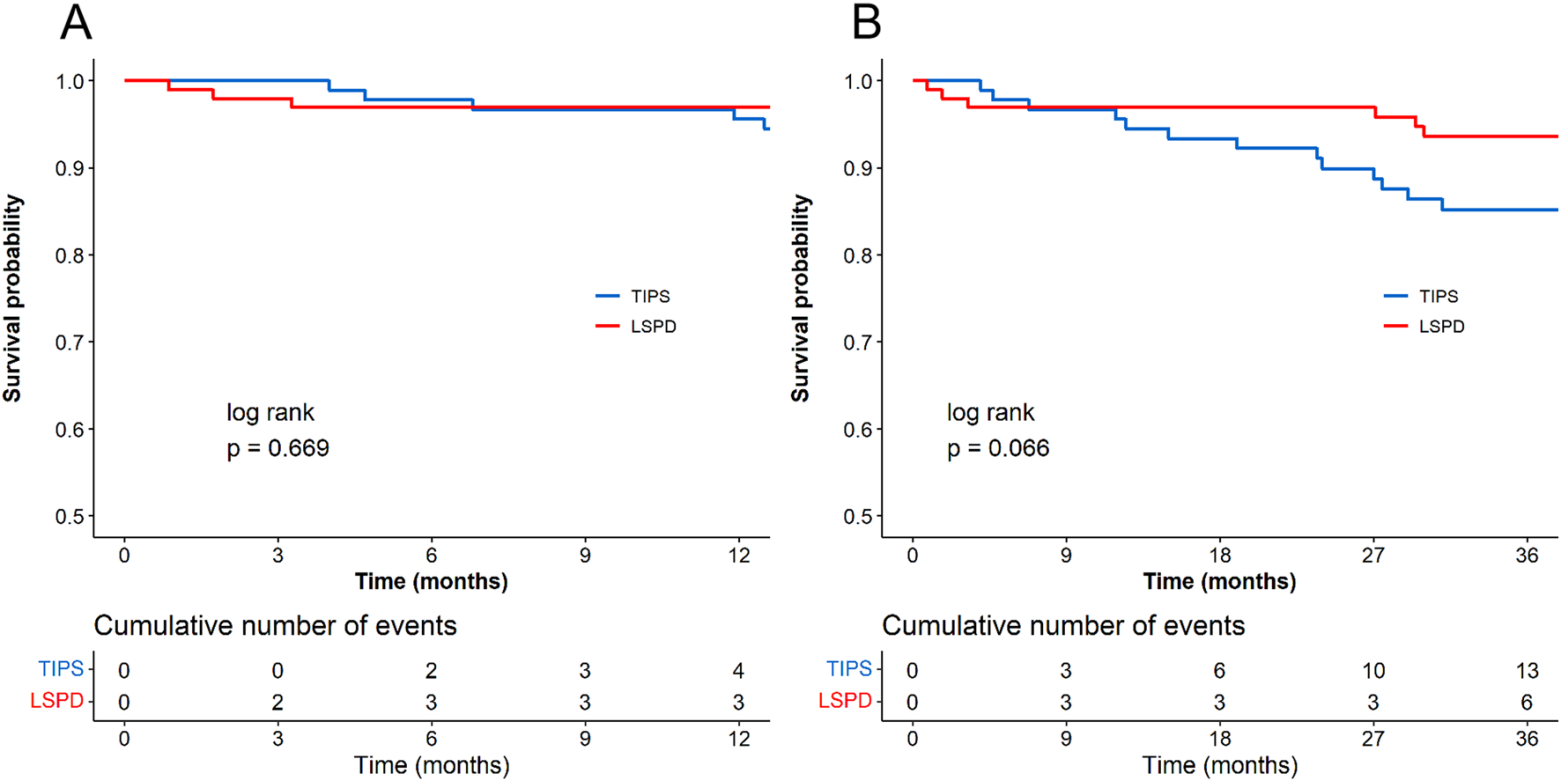
Survival curves of the two groups. Panels **A** and **B** depict the comparison of survival changes at 1 year and 3 years after surgery

The survival rates during the follow-up period were 86.60% for the TIPS group and 88.46% for the LSPD group. In the follow-up period, there were 13 deaths in the TIPS group, including 5 due to rebleeding, 2 due to hepatic encephalopathy, 1 due to splenectomy for splenic hyperfunction after TIPS, and 5 due to liver failure (Table 2). The LSPD group had a total of 12 deaths, including 4 due to rebleeding, 2 due to newly developed liver cancer, 1 due to post-fracture surgery, and 5 due to liver failure (Table 2). No significant differences in 1-year and 3-year survival rates between the TIPS and LSPD groups (95.60% vs 96.93%, *P* = 0.669; 85.20% vs 93.65%, *P* = 0.066) (Fig. 2) were noted. There were no significant differences in 1-year and 3-year survival rates after surgery for Child–Pugh A patients between the TIPS and LSPD groups (100% vs 95.55%, *P* = 0.242; 93.10% vs 93.04%, *P* = 0.967). For Child–Pugh B patients, there was no significant difference in 1-year survival rate after surgery (93.44% vs 98.10%, *P* = 0.247), but the LSPD group had a higher 3-year survival rate compared to the TIPS group (81.17% vs 94.19%, *P* = 0.041).

**Table 2:** Primary postoperative outcome

Outcome	TIPS (*n* = 97)	LSPD (*n* = 104)
Duration of follow-up (days)	1138 (870, 1398)	1715 (1236, 2295)
Rebleeding, (%)	17 (17.5)	17 (16.3)
Overt hepatic encephalopathy, (%)	23 (23.7)	3 (2.9)
Death, (%)	13 (13.4)	12 (11.5)
*Cause of death, (%)*		
Liver failure	5 (5.2)	5 (4.8)
Rebleeding	5 (5.2)	4 (3.8)
Liver cancer	0 (0)	2 (1.9)
Encephalopathy	2 (2.1)	0 (0)
Fracture	0 (0)	1 (1.0)
Post-splenectomy	1 (1.0)	0 (0)
Severe complications, (%)	31 (32.0)	36 (34.6)
Rebleeding	12 (12.4)	14 (13.5)
Overt hepatic encephalopathy	5 (5.2)	1 (1.0)
Liver failure	5 (5.2)	9 (8.7)
Liver cancer	0 (0)	4 (3.8)
Splenic hyperfunction	3 (3.1)	0 (0)
Splenic abscess	1 (1.0)	0 (0)
Stent occlusion	4 (4.1)	0 (0)
Stents stenosis	1 (1.0)	0 (0)
Postoperative abdominal bleeding	0 (0)	1 (1.0)
Postoperative abdominal infection	0 (0)	2 (1.9)
Acute intestinal obstruction	0 (0)	1 (1.0)
Pulmonary embolism	0 (0)	1 (1.0)
Pulmonary infection	0 (0)	1 (1.0)
Fracture	0 (0)	1 (1.0)
Lower limb edema	0 (0)	1 (1.0)

### Variceal rebleeding

**Fig. 3:**
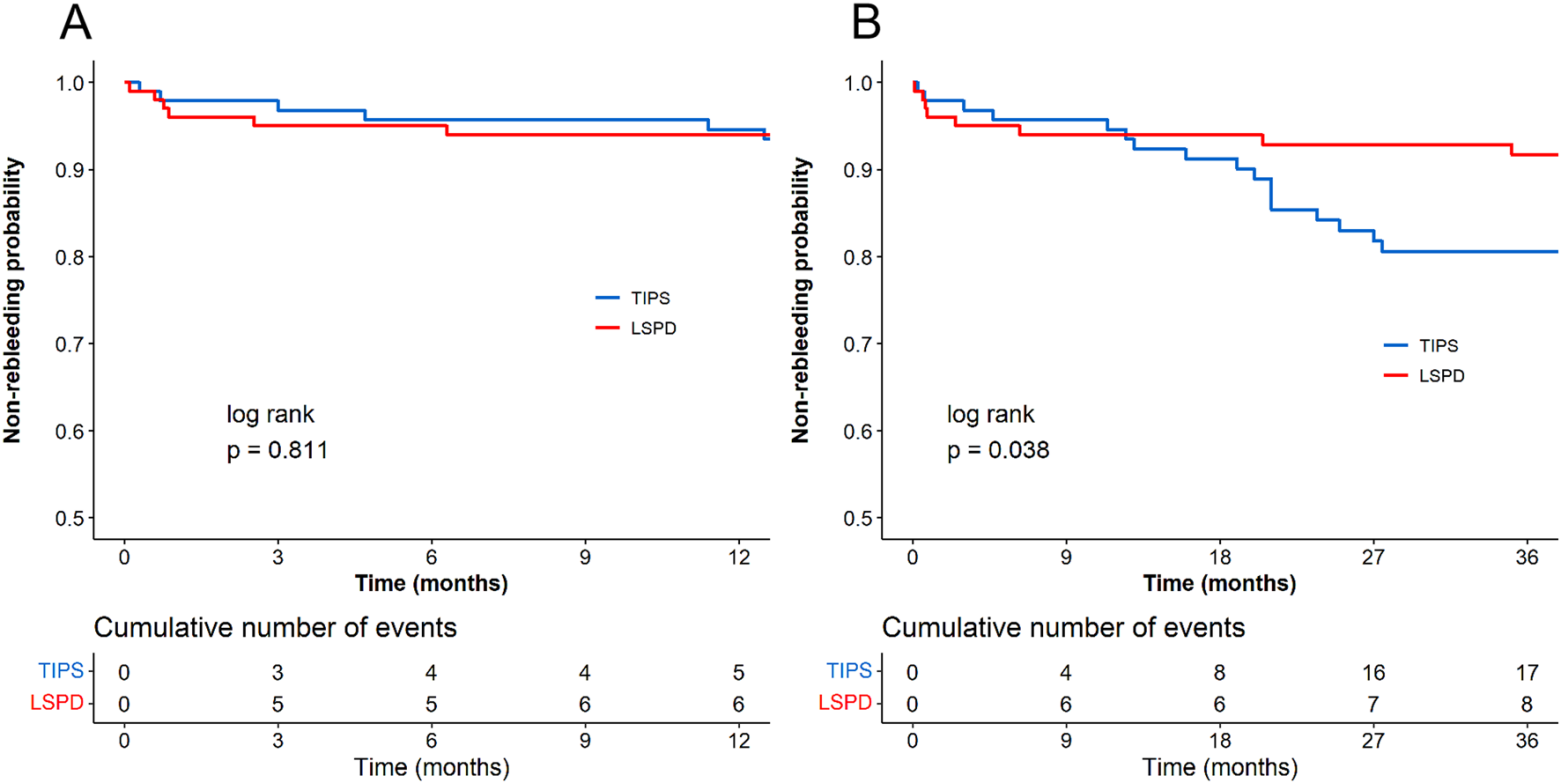
Non-rebleeding curves of the two groups. Panels **A** and **B** depict the comparison of 1-year and 3-year non-rebleeding rates

The follow-up non-rebleeding rates for the TIPS and LSPD groups were 82.47% and 83.65%, respectively. The TIPS group had 17 cases of rebleeding, resulting in 5 deaths. Among them, 4 cases received TIPS replacement due to bleeding caused by stent occlusion, 1 case underwent endoscopic treatment and stent dilation for bleeding caused by stent stenosis, and 7 cases achieved successful bleeding control through medication and endoscopy. In the LSPD group, there were 17 cases of rebleeding, resulting in 4 deaths. Two cases received TIPS treatment, 1 case underwent liver transplantation, and the remaining 10 cases achieved successful bleeding control through medication and endoscopy. The 1-year and 3-year non-rebleeding rates after TIPS were 94.62 and 80.56% and 94.00% and 91.71% for the LSPD group, respectively. No significant difference was observed in the 1-year non-rebleeding rate between the two groups (*P* = 0.811), but the LSPD group had a higher 3-year non-rebleeding rate (*P* = 0.038) (Fig. 3). There were no significant differences in the 1-year and 3-year non-rebleeding rates between the TIPS and LSPD groups among Child–Pugh A patients (100% vs 95.49%, *P* = 0.238; 85.30% vs 93.04%, *P* = 0.363), as well as among Child–Pugh B patients (91.99% vs 92.50%, *P* = 0.955; 78.16% vs 90.41%, *P* = 0.087).

### Overt hepatic encephalopathy (OHE)

**Fig. 4:**
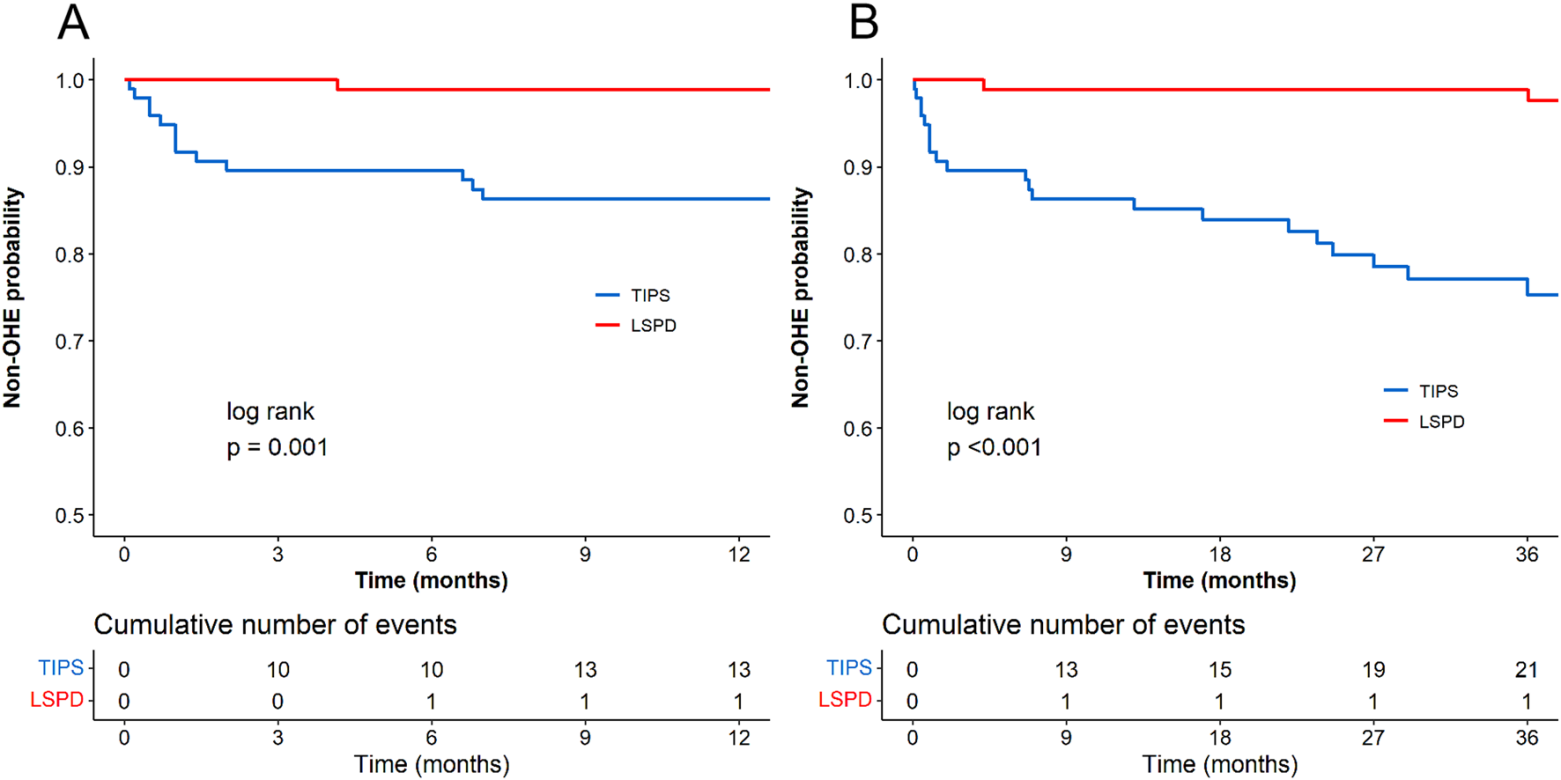
Non-OHE curves comparing of the two groups. Panels **A** and **B** depict the comparison of 1-year and 3-year non-OHE rates

The follow-up non-OHE rates in TIPS and LSPD groups were 76.29 and 97.12%, respectively. The TIPS group had 23 cases of OHE, with 2 resulting in deaths, while the remaining 21 cases were controlled through medication. In the LSPD group, there were 3 cases of OHE, all of which were controlled through medication. The LSPD group had a higher 1-year and 3-year rate of non-OHE compared to the TIPS group (98.9% vs 86.28%, *P* = 0.001; 98.9% vs 75.28%, *P* < 0.001) (Fig. 4). In Child–Pugh A patients, the LSPD group had a higher 1-year and 3-year rate of non-OHE (100% vs 90.25%, P = 0.037; 100% vs 90.25%, *P* = 0.037), as well as in Child–Pugh B patients (97.96% vs 84.12%, *P* = 0.013; 97.96% vs 67.86%, *P* < 0.001). Apparently, OHE primarily occurs in patients after TIPS procedure.

### Severe complications

**Fig. 5:**
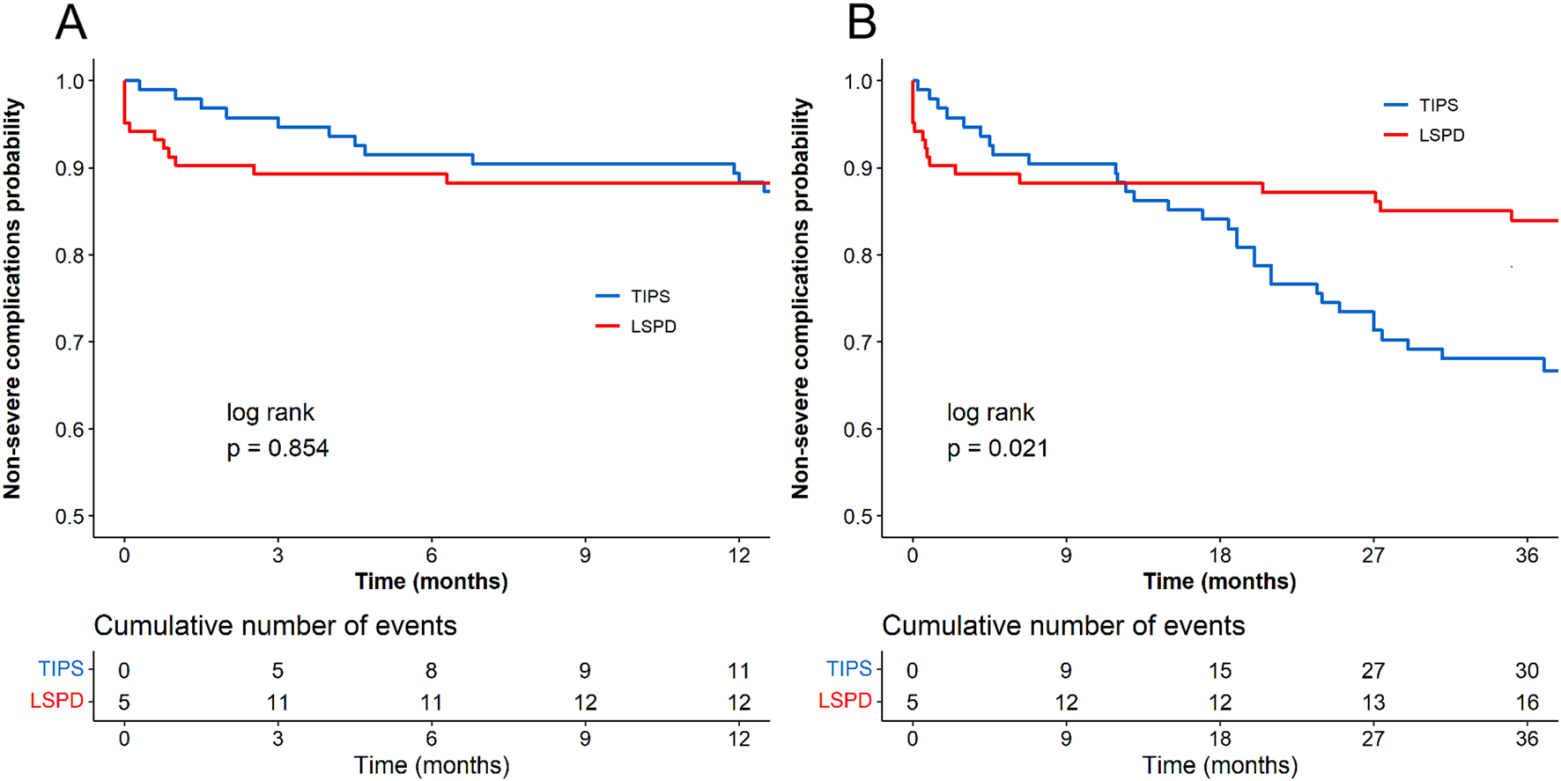
Non-severe complications curves comparing of the two groups. Panels **A** and **B** depict the comparison of 1-year and 3-year non-severe complications rates

During the follow-up period, there were 31 cases of severe complications in the TIPS group and 36 cases in the LSPD group (Table 2). The rates of non-severe complications in the TIPS and LSPD groups were 68.04% and 65.38%. There was no significant difference in the rates of non-severe complications at 1 year between the TIPS and LSPD groups (89.38% vs 90.08%, *P* = 0.950), but the TIPS group had a lower rate of non-severe complications at 3 years compared to LSPD the group (68.97% vs 83.94%, *P* = 0.020) (Fig. 5). In Child–Pugh A patients, no statistical difference in the rates of non-severe complications were observed at 1-year and 3 years after surgery between the TIPS and LSPD groups (93.55% vs 89.16%, *P* = 0.495; 82.24% vs 74.19%, *P* = 0.493). In Child–Pugh B patients, there was no statistical difference in the rates of non-severe complications at 1 year after surgery between the TIPS and LSPD groups (87.34% vs 90.8%, *P* = 0.622), but the LSPD group had a higher rate of non-severe complications at 3 years compared to the TIPS group (85.08% vs 66.4%, *P* = 0.025). Patients with Child– Pugh B in the TIPS group are more likely to develop severe complications.

## Discussion

The findings of this study demonstrate that the use of LSPD and TIPS for secondary prevention in patients with GOV bleeding is safe and effective. In our study, there was no difference in overall survival at 3 years between the TIPS and LSPD groups. However, in Child–Pugh Class B patients, the LSPD group had a higher survival rate at 3 years. The TIPS group had a higher rebleeding rate at 3 years compared to the LSPD group, and TIPS patients were more prone to develop hepatic encephalopathy.

In this study, nearly 40% of deaths were associated with organ failure caused by end-stage liver disease. Decompensation is a significant risk factor for mortality in patients with cirrhosis. Previous research has shown that the mortality risk for decompensated cirrhosis patients is 10 times higher than the general population, with in-hospital mortality rates ranging from 10 to 20% [14, 15].

Furthermore, changes in hemodynamics after TIPS procedure are important influencing factors for acute liver failure. Conservative treatment can usually alleviate this condition, and selecting smaller TIPS stents based on preoperative liver function grading can also reduce the risk of acute liver failure induced by sudden hemodynamic changes [16, 17]. Both LSPD and TIPS can reduce bleeding events caused by portal hypertension, but bleeding due to portal hypertension remains a significant cause of mortality in patients. This study showed a significant reduction in long-term rebleeding rates after LSPD. This is attributed to the interruption of gastric collateral circulation by LSPD, reducing splenic venous reflux and decreasing portal vein blood flow, thereby lowering portal vein pressure and reducing the likelihood of rebleeding [18, 19]. Patients who experience rebleeding after LSPD should consider endoscopic or TIPS therapy in addition to medication [20]. A propensity-matched study indicated that the use of incompletely expanded TIPS in patients with a history of splenectomy is safe and effective for treating post-splenectomy rebleeding [20]. However, it is important to note that placing TIPS after splenectomy may increase the difficulty due to portal vein system thrombosis and stenosis [21]. Therefore, detailed preoperative evaluation and preparation with ultrasound, angiography, and other methods are necessary for these patients before undergoing TIPS placement. In this study, the rebleeding rate after TIPS at 3 years was 19.44%, and these patients were effectively controlled through medication, endoscopy, or stent revision. The treatment for rebleeding after TIPS should be selected based on the underlying cause. Endoscopic therapy can be used for acute bleeding, while stent dilation or repositioning can be considered for bleeding caused by stent stenosis or occlusion.

Hepatic encephalopathy is a major complication following TIPS procedure. The occurrence rate of hepatic encephalopathy in the first year after the procedure was 13.72% in this study. Reported rates of hepatic encephalopathy one year after TIPS range from approximately 10% to 50% [22]. The main cause of post-TIPS hepatic encephalopathy is the direct entry of ammonia from the intestines into the brain, as it cannot be converted by the liver. This leads to excessive activation of astrocytes, astrocytic edema, and a decrease in their detoxification capability, ultimately resulting in hepatic encephalopathy [23]. Additionally, TIPS placement can cause liver parenchymal damage, vascular occlusion, or compression, leading to hepatic ischemia and subsequent hepatic encephalopathy [24]. Currently, preventive measures for post-TIPS hepatic encephalopathy mainly include optimizing stent size, optimizing portal-systemic shunt gradient, reducing spontaneous portosystemic shunting, and medication prevention. Studies on the relationship between stent size optimization and prevention of hepatic encephalopathy have yielded different conclusions. Among the more extensively studied stent sizes, 8 mm and 10 mm stents are commonly considered. Randomized controlled trials indicate that an 8-mm stent has better preventive effects on hepatic encephalopathy compared to a 10 mm stent, coupled with improvements in liver function [25, 26]. A meta-analysis involving 489 patients yielded similar results, suggesting that although the use of an 8-mm stent did not reduce the incidence of rebleeding, it had better preventive effects on post-TIPS hepatic encephalopathy. Therefore, an 8-mm stent is recommended as the preferred choice for TIPS placement [27]. Controlling spontaneous portosystemic shunting can effectively reduce the flow of blood directly from the portal vein system to the systemic circulation and lower the shunt volume. This can decrease the occurrence of post-TIPS hepatic encephalopathy to a level similar to that of patients without spontaneous portosystemic shunting. Hence, it is recommended to perform TIPS placement in conjunction with variceal embolization [28].

Excluding severe complications, such as death, rebleeding, hepatic encephalopathy, liver failure, and stent occlusion, other serious complications in the TIPS group mainly included stent occlusion without bleeding or death, splenic hyperfunction, splenic abscess, and splenic rupture. In the LSPD group, complications included secondary liver cancer, intestinal obstruction, and pulmonary infection. These patients showed varying degrees of improvement after different treatments without causing death. For patients with splenic hyperfunction after TIPS, combined treatment with splenic artery embolization is recommended, with an embolization area of 50 to 70% [29]. Studies have shown that when the embolization area is less than 50%, the embolization effect is not significant, while an embolization area greater than 70% increases the incidence of complications, particularly the formation of splenic abscess, which carries a high risk when exceeding 70%. An embolization area of 50 to 70% represents a balance between therapeutic efficacy and complications following splenic artery embolization. In cases where interventional embolization is contraindicated, laparoscopic splenectomy may be considered [30].

Due to the rarity of performing TIPS or LSPD in Child–Pugh C patients, this study did not compare the efficacy in Child–Pugh C patients. In our study, there were a total of 10 Child–Pugh C patients in both groups (2 in the TIPS group and 8 in the LSPD group). Preoperative data analysis revealed that both groups had poor liver function and coagulation function, as well as moderate to severe ascites. Further follow-up found that out of these 10 patients, 6 (60%) experienced severe postoperative complications and 5 (50%) had serious complications. Therefore, although TIPS or LSPD is not strictly contraindicated in Child–Pugh C patients, preoperative medical treatment can achieve down-staging effects, but clinicians should still be vigilant about the occurrence of severe postoperative complications.

This study has certain limitations. Firstly, we did not discuss the improvement or worsening of the severity of postoperative esophageal varices or complications related to portal vein system thrombosis, due to the lack of detailed imaging and endoscopic follow-up data. Portal vein thrombosis is a common complication after LSPD, but severe complications caused by thrombosis are rare. Some studies have reported the preventive effects of different postoperative anticoagulant/antiplatelet treatments on the formation of portal vein system thrombosis, which can reduce the incidence of thrombotic events to varying degrees [31, 32]. Secondly, due to clinical preferences, there were differences in the treatment received by different patients, especially in Child–Pugh B patients, where clinicians may lean more toward choosing TIPS surgery. In addition, the initial department responsible for treating bleeding can also lead to different treatment choices. Therefore, further randomized controlled trials would help balance patient selection biases and provide a more objective evaluation of LSPD and TIPS.

In conclusion, this study demonstrates that for patients with liver function classified as Child–Pugh A and B, both TIPS and LSPD are safe methods for preventing death and rebleeding in patients with esophageal varices. Compared to TIPS, LSPD does not increase the risk of death and rebleeding, and it is a safe and effective treatment for EVB in Child–Pugh A and B patients.

### Supplementary Information

Below is the link to the electronic supplementary material.

Supplementary file1 (DOCX 14 KB)
