# Supplementary material for: Prognosis of LSPD versus TIPS for the treatment of esophagogastric variceal bleeding in cirrhosis
Source: Surg Endosc. 2024 Mar 4;38(4):2106–15. doi: 10.1007/s00464-024-10729-7 (PMC10978701; doi:10.1007/s00464-024-10729-7)
Supplement: Supplementary file 1 — Supplementary file1 (DOCX 14 KB) [file 464_2024_10729_MOESM1_ESM.docx]

**Surgical procedure**

**TIPS**

Materials

TIPS Coated Stent System (GORE® VIATORR® TIPS Endoprosthesis) (Gore, USA), Vascular Sheath Set (Radifocus) (Terumo Co., Ltd., Japan), RUpS-100 Penetration Kit (COOK, USA), Multi-Purpose Catheter (Johnson & Johnson, USA), Embolic Spring Coil (COOK, USA), Microcatheter (Terumo Co., Ltd., Japan), Yashiro Catheter (Terumo Co., Ltd., Japan), 300-500μm PVA Microspheres (Alicon Pharmaceutical Co., China).

Procedure

1. Internal jugular vein puncture and imaging: The right internal jugular vein is punctured using the Seldinger technique. A guide wire, 10F long sheath, 5F Cobra catheter, multi-purpose catheter, 5F pigtail catheter, and microcatheter are inserted. Contrast medium is injected for hepatic vein angiography to observe its anatomical features.
2. Portal vein puncture: The guide wire is removed, and a TIPS puncture kit is used to enter the hepatic vein. After adjusting the direction under fluoroscopy, the liver is punctured to access the left or right branch of the portal vein. Contrast medium is injected to confirm successful puncture and observe portal vein thickening and esophageal varices.
3. Placement of covered stent system: The puncture needle is removed, and a 8 mm TIPS covered stent system is introduced into the portal shunt channel. Balloon catheter dilation and stent attachment are performed, followed by angiography to assess blood flow.

**LSPD**

Materials

Laparoscopic equipment (Karl Storz, Germany), Endoscopic pneumoperitoneum machine (Karl Storz, Germany), Ultrasonic knife mainframe (Anhe, China), Ultrasonic Knife (Anhe, China), Laparoscopic arthroscopic head linear cutting anastomosis and staple compartment (Johnson & Johnson, USA), Hemo-lock clip (Wedu Medical Co., China), Trocar (Johnson & Johnson, USA).

Procedure

Splenectomy

1. Open the greater curvature of the stomach along the gastric colonic ligament using an ultrasonic knife, exposing the tail of the pancreas and the hilum of the spleen.
2. Separate and expose the splenic artery and locally free it with an ultrasonic knife. Then use Hemo-lock clips to ligate the main trunk of the splenic artery. When the splenic artery is densely adhered to the splenic vein or surrounding tissues or there are anatomical variations, avoid forced separation. Instead, use a 2-0 Prolene suture to ligate the splenic artery to prevent major intraoperative bleeding.
3. Continue to free the spleen to the splenic hilum, including the splenocolic ligament, splenogastric ligament, splenorenal ligament, and phrenosplenic ligament. Then expose the weak point of the remaining splenogastric ligament, suspend the hilum of the spleen with a size 7 silk thread, and use bipolar electrocoagulation forceps to achieve hemostasis on the wound, reducing intraoperative blood loss.
4. Use a cutting stapler to cut and close the splenic hilum, taking care to select an appropriate staple size based on the thickness and width of the hilum. Bipolar electrocoagulation forceps are used to achieve hemostasis on the incision, and careful inspection is performed to ensure no obvious bleeding points.
5. Use an ultrasonic knife and bipolar electrocoagulation forceps to completely free the splenic ligaments and adhesions. Place the spleen in a specimen bag, crush it, and then remove it through an enlarged puncture hole. Finally, close the incision.

Dissection of the vessels around the cardia

1. Re-establish pneumoperitoneum and suspend the gastric body. Dissect and transect the short gastric vein, posterior gastric vein, and left inferior phrenic vein. Free the tissues surrounding the left gastric artery and coronary vein, use an EC60 cutting stapler to cut and close them, and apply Hemo-lock clips to clamp and reinforce the residual ends, ensuring no obvious bleeding at the transection site.
2. Free the tissues around the esophagus along the cardia and transect the gastric branches, esophageal branches, high-positioned esophageal branches, and ectopic high-positioned esophageal branches of the coronary vein until approximately 8cm from the cardia.
3. Check for any obvious bleeding on the incision, use bipolar electrocoagulation forceps to achieve hemostasis on the bleeding points, and place a drainage tube in the splenic fossa. Appropriate hemostatic materials can be sprayed on the incision to reduce the possibility of postoperative bleeding. Finally, use an abdominal wall closure device to close the Trocar hole.
